# Functional bioactive compounds in ginger, turmeric, and garlic

**DOI:** 10.3389/fnut.2022.1012023

**Published:** 2022-12-08

**Authors:** Christiana Oluwatoyin Ajanaku, Olabisi Theresa Ademosun, Prudence Osahenomanse Atohengbe, Samuel Oluwakayode Ajayi, Yemisi Dorcas Obafemi, Olayinka Ayotunde Owolabi, Paul Akinniyi Akinduti, Kolawole Oluseyi Ajanaku

**Affiliations:** ^1^Department of Chemistry, Covenant University, Ota, Ogun State, Nigeria; ^2^Department of Biological Sciences, Covenant University, Ota, Ogun State, Nigeria; ^3^Department of Agriculture Economics and Extension, Landmark University, Omu-Aran, Kwara State, Nigeria

**Keywords:** ginger, garlic, nutraceuticals, nutrition, therapeutics, turmeric

## Abstract

Nutrition plays a very important role in the health promotion of individuals and brought about a global paradigm shift from pharmaceuticals to nutraceuticals. This is due to the high cost, non-availability, and side effects associated with the unregulated consumption of pharmaceuticals. Over the ages, nutraceuticals from food products were reported to contain bioactive compounds with great health and physiological benefits. This report reviews bioactive compounds in selected foods namely ginger (*Zingiber officinale*), turmeric (*Curcuma longa*), and garlic (*Allium sativum*) as potential natural therapeutics for ailments of cancer and heart-related diseases. Analytical profiles, functional activities, and characterization of these compounds were discussed with possible recommendations for the prospective treatment of diseases using these nutraceuticals.

## Introduction

**Figure 1:**
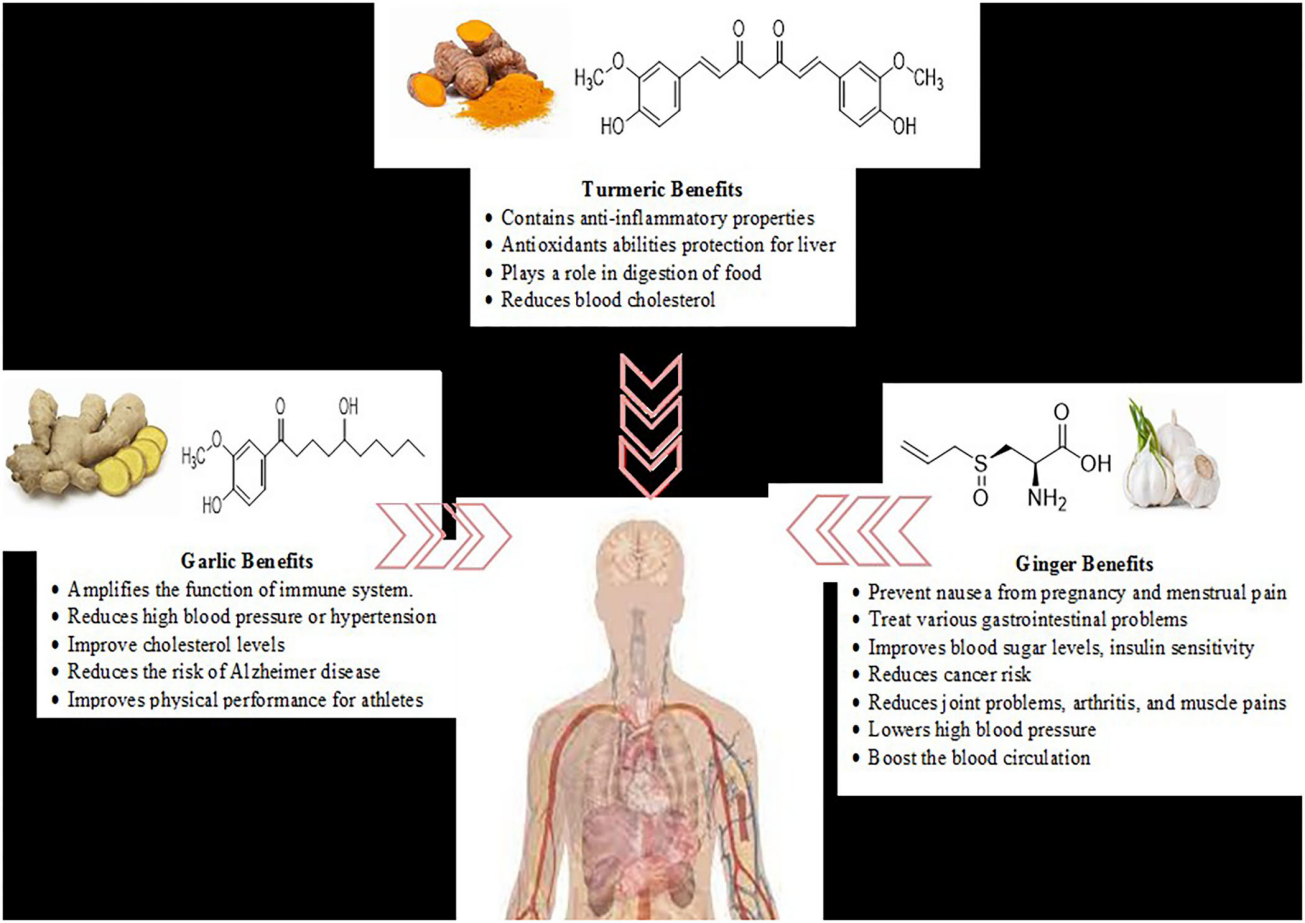
Health-associated benefits of ginger (*Zingiber officinale*), turmeric (*Curcuma longa*), and garlic (*Allium sativum*) as potential nutraceuticals.

Nutraceuticals are nutritional products that help to improve health and prevent disease. They include a large number of products, dietary supplements, nutrients, herbal products, and specific processed food and beverages (1). These products are mostly plant-based and some animal-based foods, and omega fatty acids. Bioactive compounds obtained from herbs, fruits, foods, and spices were used for culinary purposes and consumption (2). Fortified foods are categorized chemically as: carbohydrate derivatives (oligosaccharides, non-starch polysaccharides), fatty acids, structural lipids (mono and polyunsaturated fatty acids), derivatives of isoprenoid (terpenoids, saponins, carotenoids, terpenes and all forms of vitamin E such as tocopherols, tocotrienols), phenolic compounds and vitamin C, for the benefit of humankind and nature (3). Bioactive compounds present in natural products, foods, and fruits exert pharmacological effects and therefore add to the functionality of foods (4). This review detailed the potential health-associated benefits of bioactive compounds obtained from selected plant-based food products including ginger, turmeric, and garlic were presented in Figure 1.

## Functional nutraceutical components in ginger (*Zingiber officinale*)

Ginger (*Zingiber officinale*) is a tropical flowering plant that was originally cultivated in southeastern Asia but has now been widely planted and distributed around the world. It is classified as a member of the Zingiberaceae family that produces a cluster of greenish-purple flowers about three feet tall. The root is called a rhizome and has been used partly as a flavor. The ginger flowering plants contain a lot of varieties of around 47 genera, ≥1,000 species with different floral arrangements, and sizes of the rhizome. Some of the varieties of ginger include Crepe Ginger (*Costus speciosus*), Kahili Ginger (*Hedychium gardnerianum*), Pineapple Ginger (*Tapeinochilos ananassae*), Torch Ginger (*Etlingera elatior*), and White Ginger (*Hedychium coronarium*) (5, 6).

The annual production of ginger was reported to be about 3.3 million tons in India which accounted for 34% of the global garlic production as shown in Table 1 (6). The ginger rhizome contains 60–70% carbohydrates, 3–8% crude fiber, 9% protein, 8% ash, 3–6% fatty oil, and 2–3% volatile oil, phenolic compounds, and terpenes (7, 8). The terpene components in ginger include zingiberene, beta-bisabolene, alpha-farnesene, beta-sesquiphellandrene, and alpha-curcumene, while phenolic compounds include gingerol, paradol, and shogaol. These gingerols and shogaols are of higher concentration than other compounds (9).

**Table 1:** Estimated global production of ginger (*Zingiber officinale*).

**Country**	**Production (tons)**
India	1,109,000
Nigeria	522,964
China	463,707
Indonesia	340,341
Nepal	271,863
Thailand	164,266
World	3,270,760

Source: FAOSTAT (6).

The locals in Nigeria have used ginger over the ages to prevent nausea resulting from pregnancy and menstrual pain (10). It is used to treat various gastrointestinal problems such as upset stomach, diarrhea, dyspepsia (discomfort after eating), morning sickness, bloating, heartburn, and loss of appetite (11). Ginger helps to improve blood sugar levels and insulin sensitivity and reduces cancer risk (12). Its fresh juice is also used locally as an anti-inflammatory agent that helps to reduce joint problems, osteoarthritis, rheumatoid, arthritis, and muscle pains (13). Fresh juice from ginger was also used to treat skin burns (14). The active component of ginger was used as an antacid medication and a laxative. It lowers high blood pressure and boosts the circulation of blood by warming the body (15). This warming effect enables ginger to act as an antiviral for treating flu and cold (16). Chronic inflammation and oxidative stress are key drivers of Alzheimer's disease and the cognitive decline that accompanies age (17). There are suggestions that the bioactive compounds and antioxidants present in ginger help to inhibit inflammatory responses that occur in the brain and have ameliorative effects in folk medicine for the management of various ailments (18).

Zingerone (4(4-hydroxy-3-methoxyphenyl)-2-butanone) is a volatile bioactive compound produced by the degradation of shogaols and gingerols. It consists of a phenolic ring with a methoxy group attached to a benzene ring. This compound exhibits a wide range of pharmacological activities and is relatively non-toxic (19). Zingerone is present in dried ginger and can also be obtained from gingerol through retro aldol reaction (20). Reports from the pharmacokinetic study of zingerone revealed oxidation of side chains in all available sites upon ingestion of zingerone orally or intraperitoneally. Its pharmacological activities consist of anti-cancer, antioxidant, and antimicrobial (21). Fresh ginger was reported to contain some contents of 6-gingerol, 8-gingerol, and 10-gingerol using high-profile liquid chromatography with an increased amount of zingerone upon drying or roasting (22).

Zingiberene was generated through the isoprenoid pathway from farnesyl pyrophosphate (FFP) which was rearranged to form nerolidyl diphosphate. After the pyrophosphate has been removed, the ring is closed, leaving only the tertiary carbon carbocation that is attached to the ring. A more stable allylic carbocation is then generated by the occurrence of a 1,3-hydride shift. The final step involves the removal of the cyclic allylic proton and the subsequent double bond formation. The enzyme responsible for catalyzing this reaction is known as zingiberene synthase and is used in forming other mono- and sesquiterpenes (23).

## Nutritional attributes of turmeric (*Curcuma longa*)

Turmeric (*Curcuma longa*) is a flowering plant used as a condiment that belongs to the family *Zingiberaceae*. The plant grows in Asia, especially in India and Central America. A source of red spice originating from plants which enables it to act as a coloring agent, flavoring agent, and as ingredient for curry powder. Turmeric is of different varieties including Suvana, Sudharsana, Prabha, Suguna, Pragati, Kedaram, and Prathibha. Turmeric is added as a supplement to teas, powder-containing capsules, and extracts (24).

The United State Department of Agriculture (USDA) National Nutrient Database stated that one tablespoon of turmeric powder contains: 29 calories, protein (0.91 g), fat (0.31 g), carbohydrate (6.31 g), fiber (2.1 g), sugar (0.3 g), iron (16%), potassium (5%), vitamin C (3%), and manganese (26%) based on daily human requirements (25). Turmeric contains phytochemical components including diarylheptanoids and curcuminoids such as curcumin, dimethoxycurcumin, and bisdimethoxycurcumin (26, 27). The compounds Turmerone, germacrone, zingiberene, and atlantone generate the essential oils present in turmeric (28).

Turmeric was used in Ayurvedic medicine for different health issues (29). Turmeric containing anti-inflammatory properties helps to reduce the pain of people who are suffering from arthritis. Turmeric has antioxidant abilities that help to protect the liver from being damaged by toxins (30). It adds flavor to food and spice and plays a role in the digestion of food (31). It is an effective aid in reducing blood cholesterol (32). Other possible uses include treatment for cancer, pre-diabetes, tuberculosis, and Alzheimer's disease (33). However, pregnant women should avoid taking turmeric supplements because of their blood-thinning effects (34). The stomach produces more gastric acid due to stimulations from turmeric. It could positively affect the digestive system of some people while others could be negatively affected (35, 36).

Curcumin is the principal curcuminoid found in turmeric. Curcuminoids are diarylheptanoids that consist of curcumin and its various derivatives such as demethoxycurcumin, bis-curcumin, and cyclic curcumin (37). Chemical groups are added to them to make curcumins more soluble which enhances their suitability for drug use. It is a symmetric molecule also called diferuloylmethane (38). The IUPAC name of curcumin is (1E, 6E)-1,7-bis (4-hydroxy-3-methoxyphenyl)-1,6-heptadiene-3,5-dione with chemical formula C_21_H_20_O_6_. It consists of three different chemical entities in its structure and they include two aromatic ring systems containing o-methoxy phenolic groups, connected by a seven-carbon linker that consists of an alpha,beta-unsaturated beta-diketone moiety (39).

The diketone group can form stable enols and readily undergo keto-enol tautomerism, which depending on the environment can exist as different types of conformers (40). It exists in a cis-enol configuration at its crystal state which is stabilized by resonance-assisted hydrogen bonding and the structure consists of three substituted planar groups interconnected through two double bonds. Depending on the nature of the solvent, which is mainly polar or non-polar, the enol form is generally more stabilized than the keto form. A pi electron cloud surrounds the molecule because of extended conjugation. Curcumin exists as cis-trans isomers when present in a solution form. In its trans-form, it contains two phenolic methoxy groups on the opposite side of its backbone and is more stabilized than the cis form where the phenolic methoxy groups are on the same side up the backbone. It is sparingly soluble in water and hydrocarbon solvents such as hexane but dissolves readily in polar solvents such as chloroform, acetonitrile, ethyl acetate, ethanol, and methanol (41).

Curcumin has three highly reactive functional groups namely one diketone moiety and two phenolic groups (42). It exhibits various chemical reactions that enhance its bioactivity and they include nucleophilic addition reactions that could be either reversible or irreversible, enzymatic reactions, and hydrogen transfer which leads to the oxidation of curcumin. Curcumin also forms stable complexes with metals and non-metals. It is a monobasic bidentate ligand and acts as an excellent chelating agent (43).

## Garlic (*Allium sativum*) as a potential functional nutraceutical

Garlic (*Allium sativum*) is an important plant from the family *Amaryllidaceae*. It is a specie in the onion genus *Allium*. It is grown for its flavorful bulb as shown in Figure 1. It is mainly cultivated in Asia but is also found in several places across Europe. Raw garlic has a pungent taste and its bulb releases a powerful onion-like aroma. It is consumed either as a vegetable raw material or after processing in the form of oil, extract, and even powder, with these different garlic formulations, a discrepancy in the chemical composition and consequently the bioactive content of compounds is observed (44). Garlic varieties are of two major types namely: softneck (*Alium sativum*) and hardneck (*Allium ophioscorodon*). Examples of softneck varieties include “applegate,” “Italian purple,” “California late,” “Polish Red,” “Red toch,” “California Early,” “Inchelium red,” “Galiano,” “Polish white,” and “kettle River grant,”. Examples of hardneck varieties include “Chesnok red,” “German white,” “Porcelain,” “Persian star,” and “Purple stripe” (45). The estimated global production for garlic (*Allium sativum*) was recorded at 26.6 million tons with China accounting for 80% of total production as shown in Table 2 (6).

**Table 2:** Estimated garlic (*Allium sativum*) production worldwide.

**Country**	**Production (tons)**
China	21.2
India	1.4
Bangladesh	0.38
European Union	0.3
South Korea/Egypt	0.28/0.28
Russia	0.26
World	26.6

Source: FAOSTAT (6).

Garlic is an annual crop and is propagated by planting cloves or top bulbils. The main quality feature of garlic products is the distinct flavor of cloves, because of complex biochemical reactions (46). It comes in different forms such as paste, powder, and extracts. The chemical constituents present in garlic are mainly sulfur-containing, non-volatile amino acids (thiosulfinates), which include alliin or S-allyl-cysteine, ajoene, diallyl polysulfides, vinyldithins, Alliinase, and saponins. A clove (3 g) of raw garlic contains manganese (2%), vitamin B6 (2%), vitamin C (2%), selenium (1%), fiber (0.06 g), and trace amounts of iron, vitamin B1, potassium, calcium, phosphorus, and calcium based on the human daily requirement (25). Diallyl disulfide, diallyl trisulfide, and allyl propyl sulfide are principal components of essential added to food to complement it and have medicinal properties (47). Garlic has a wide range of health benefits to the body system. It is known to amplify the function of the immune system. The active compounds in garlic help to reduce high blood pressure or hypertension and improve cholesterol levels. The antioxidants in garlic support the mechanism provided in the body to prevent health issues because of oxidative stress, Alzheimer's disease, itching, ringworm, and athlete's foot (48, 49).

Organosulfur compounds are the major bioactive compounds found in garlic and they are mainly allicin, allin, and ajoene. Allicin is an oily yellow liquid that provides the unique smell of garlic. Allicin's significance as a biologically active compound is due to its high reactivity with a low and high molecular weight of thiol-containing protein, antioxidant activity, and accessibility due to its high permeability (50). Allicin is formed by enzymatic reactions by the enzyme allinase on allin. It is activated when the bulb of garlic is crushed. The reaction begins with the conversion of cysteine into S-allyl-L-cysteine. This thioether is then oxidized to form a sulfoxide known as allin. Allinase enzyme with the aid of pyridoxal phosphate cleaves allin, and the dehydration of allin occurs. Allin is then converted into allyl sulfenic acid, pyruvic acid, and ammonia. Allyl sulfenic acid is highly reactive at room temperature and the elimination of water occurs. At room temperature, two molecules of allyl sulfenic acid condense and forms allicin (51). Allicin exists mainly as a racemate, although it is chiral. It is unstable and breaks down within 16 h at 23°C. Allicin is decomposed into diallyl disulfide, diallyl trisulfide, vinyldithins, and ajoene. These pungent compounds are metabolized to form allyl methyl sulfide. This allyl methyl sulfide takes several hours to be released to the lungs and the skin; hence, the effect of eating food-containing garlic is felt for a long time. Allicin stability and biological activity is enhanced by the formation of an inclusion complex with cyclodextrins. Ajoene which has a chemical formula C_9_H_14_OS_3_ and an IUPAC name (E)-1-(prop-2-enyldisulfanyl)-3-prop2-enylsulfainylprop-1-ene is formed from allicin decomposition and can be used for a large range of biological activities such as anticancer activity. It consists of E and Z isomers and contains sulfoxide and disulfide functional groups (52).

## Potential therapeutic properties of bioactive compounds in the selected plant-based foods

Bioactive compounds have played a crucial role in improving health and chronic disease prevention. These phenolic compounds including flavonoids, terpenes, curcumin, organosulfur compounds, and various other bioactive compounds present in food serve as antioxidants, anti-inflammatories, and anticancer. The phenolic compounds such as flavonoids present in small quantities in garlic suppress the synthesis of reactive oxygen species, inhibit enzymatic action, and act as chelating agents to prevent the production of free radicals. It was also used to improve antioxidant defenses and the scavenging of reacting oxygen species. The antioxidant activity of these flavonoids is due to their functional group conformational disposition and it involves their configuration, substitution, and the total number of hydroxyl group present (53).

Ginger was discovered to contain active ingredients that combat degenerative, allergic, metabolic, and cardiovascular disorders. Ginger has also been suggested as an anti-diabetic agent; however, it is required that its active constituent's anti-diabetic, anti-hyperlipidemic, and anti-hyperglycemic effects should be assessed. Gingerols present in ginger enhanced the ingestion of glucose in adipocytes of the skeletal muscle (54). This uptake was attained by the rise of GLUT4 expression on the cell membrane and the AMP-activated protein kinase (AMPK) pathway becoming activated leading to the reversion of hyperglycemia and other abnormal metabolic condition from diabetes. The bioactive compounds including the gingerols, shogaols, and paradols showed greater anti-hypoglycemic activity due to the presence of unbranched alkyl chains, unlike zingerone which showed limited activity with various functional pathways affected by these bioactive compounds. In addition, shorter side chains shogaols enhance glucose utilization, and longer chain shogaols impede the accumulation of lipids in adipocytes (55). Paradols are reported to have anti-obesity activities that may be enhanced with increasing chain length through thermogenesis activation in brown adipose tissue in the body (55).

Zingerone has the ability to scavenge reactive oxygen species, free radicals, peroxides, and other oxidants. This allows for high-potential therapeutic candidates for the treatment of various diseases such as Alzheimer's disease, atherosclerosis, and Parkinson's disease (56). Xanthine oxidase functions by producing free radical that causes oxidative damage and zingerone has been discovered to protects against stannous chloride-induced and hydrogen peroxide-induced oxidative DNA damage *in vitro*. (56). Zingerone also acts as an antidiarrheal by modification of bacteria and the various metabolism in the cell. Ginger supplements aided in the prevention of nausea generated due to chemotherapy (56, 57).

Curcumin extracted from turmeric was reported to possess scavenging activity on reactive oxygen species through the transfer of electrons and protons from the phenolic group. The extended conjugation of curcumin stabilizes the phenoxyl radicals generated by this reaction. The pleiotropic activity of curcumin enables it to interact with the various molecules in the cell, which include DNA, metals, metalloproteins, proteins, and lipids. The phenolic moiety, the keto-enol tautomeric group, and the carbon linkage functional group of curcumin are all involved in various covalent and non-covalent interactions of curcumin with biomolecules. This carbon linkage helps curcumin become more flexible for more productive hydrophobic interaction (58). Curcumin acts as an anti-tumor through its interactions with nucleic acids. From reviews, using yeast RNA and calf thymus DNA, through hydrophobic interactions or hydrogen bonding, curcumin and its derivatives such as dimethoxy curcumin and diacetyl curcumin could bind DNA and RNA. Over the years, there have been many reviews on curcumin metal complexes (58, 59). Curcumin forms stable complexes with most metals and non-metals and as such acts as a chelating agent. The enolic group forms this complex as the proton is substituted by the metal ion. The stability and reactivity of the complexes depend on the metal ion nature as well as the stoichiometric conditions of the reactions and these determine the structure and physical properties of the complexes. Curcumin metal complexes decrease toxicity and they affect the biological reactivity of metals, thereby creating a form of metal-based antioxidants. Alzheimer's disease was found to be greatly curtailed by the regular consumption of curcumin. This was observed by curcumin's lipophilic ability that enabled it to permeate the brain barrier and decrease toxicity in the neurons by chelating toxic metal ions present (59).

Reports indicated that curcumin exhibits anti-inflammatory properties through different mechanisms that involve the reduction of inflammatory transcription factors, redox status, and protein kinases in the nucleus. Curcumin was also reported to help in reducing knee and joint pains for patients of osteoarthritis resulting in the use of curcumin to reduce the circulation of cytokines in the body such as interleukin-6 that play a role in inflammatory pain (60). Curcumin-containing nutraceuticals were also reportedly given to patients with metabolic diseases and were discovered to improve the serum lipid levels of these patients. Curcumin-containing nutraceuticals lower the circulation of C-reactive protein that serves as a biomarker for predicting cardiovascular disease (61). Curcumin and various curcuminoids help to treat various skin diseases with their antimicrobial, antioxidant, anti-inflammatory, and various other wound healing activities (61).

Organosulfur compounds are abundant major bioactive compounds present in garlic. These sulfur-containing compounds such as allin, allicin, ajoene, vinyldithins, and ally sulfides are responsible for its flavor, but they also provide garlic with medicinal properties such as anticancer, antioxidant, antimicrobial, anti-inflammatory, cardioprotective, immunomodulatory, and antidiabetic activities (62). Garlic generally possesses strong defensive mechanisms against various pests and pathogens due to the volatile nature of the bioactive compounds. There were reports on the bioactive properties of organosulfur compounds: Ajoene and ally sulfides contained anticancer compounds along with allicin that obstructs the release of cytokines C by mitochondria, inhibit nitrosamines bioactivation, and serve as a blockage for the proliferation of cancer cells (62). Organosulfur compounds contained antioxidant properties involved in activating radicals, generating interactions with thiol-containing proteins, scavenging hydroxyl radicals, modification of SH-dependent activities, and obstructing the generation of superoxide and NO radicals. Cardio-protective properties were found in ajoene and allicin involving platelet aggregation obstruction, lowering of blood pressure, lipid profile alteration, vasodilatation enhancement, and inhibition of cholesterol biosynthesis (63).

## Extraction methods for bioactive compounds in selected plant-based foods

Over the years, ginger was extracted from its rhizome and various active components have been obtained by various methods. The reported extraction methods that gave insights into the physicochemical properties, molecular weight, and antioxidant activity of ginger include ultrasonic-assisted extraction and hot water extraction (64). For example, ginger pomace polysaccharides were characterized by using High-performance Gel Filtration Chromatography (HPGFC), High-Pressure Liquid Chromatography (HPLC), ultraviolet spectra, and infrared spectra purposes (65). The Kjeldahl method, dinitrosalicylic acid colorimetric method, ambient pressure drying, and soxhlet extraction methods were used to obtain protein content, total sugar content, water, and lipid content, respectively. The Folin–Ciocalteu method was used to determine the phenolic compounds while the aluminum chloride colorimetric method was used for flavonoid compounds. The main phenolic concentrations such as 6-gingerol, 8-gingerol, 10-gingerol, and 6-shogaol were obtained by HPLC. The mobile phase used in this separation involved two solvents that were water and acetonitrile at column temperature maintained at 30°C and 280 nm wavelength as well as HPLC, NMR, or GC/MS method. This HSCCC method generated an extremely pure concentration of 6-gingerol, 8-gingerol, and 10-gingerol and saved less time compared to conventional methods of extraction (66).

Ginger oleoresin and volatile oils were extracted by solvent extraction and steam distillation. The techniques of extraction required multiple unit operations and involved high temperatures, which leads to degradation in its active compounds and yield. Supercritical CO_2_ can extract compounds without introducing harmful chemicals and there is no form of degradation by heat. Supercritical CO_2_ can be used to isolate and characterize the various volatile oils present in ginger by fractional separation. Extraction and characterization of 6, 8, 10 gingerols, and 6-shogaols, using supercritical CO_2_ and HPLC revealed a higher yield of gingerols, zingerones, and alpha-zingiberene active compounds and quality oleoresin in comparison to conventional soxhlet extraction methods (19). Reflux extraction of various active compounds from ginger has been carried out through the process of agitated extraction, ultrasonic and microwave micellar extraction of Zingerone, gingerol, paradol and shogaol coupled with Ultra-High Performance Liquid Chromatography-UV as detector (UHPLC-UV) (67). Comparison between the microwave-assisted micellar extraction with ultrasonic-assisted extraction, soxhlet extraction and other methods revealed that the microwave-assisted micellular extraction coupled with UHPLC-UV detector had a short extraction time and the use of deoxycholic acid sodium salt in aqueous solution is environmentally friendly when compared with organic solvents.

Curcumin, the major bioactive compound generated from turmeric, has been extracted by various means from dried turmeric rhizome (68). The solvent then passes through distillation where oleoresin containing 25–35% coloring matter is yielded with volatile oil and other resinous extracts. The oleoresin is further washed by selective solvents that extract the curcumin pigment from the oleoresin and produces 90% coloring matter content along with a little volatile matter and other dry matter. Common solvents used include isopropanol, ethyl acetate, ethanol, methanol, acetone, and carbon dioxide (69).

The extraction of curcumin oil from turmeric is by distilling fresh rhizomes and leaves with Clevenger-type apparatus for 4 h; the oil obtained is passed through anhydrous sodium sulfate to ensure the oil is dried. Analysis of these oils was either carried out by Gas Chromatography at a temperature range of 70–240°C using helium, nitrogen, or carbon dioxide for separation and with a flame ionization detector (FID) for detection (70). The volatile components were detected by comparison of their retention indices. Chlorinated solvents cannot be used for the extraction of turmeric, as they are deemed harmful to food. Other methods of extracting curcumin such as dipping methods, zone-refining methods, microwave extractions, soxhlet, and ultrasonic extraction. It was concluded that soxhlet, ultrasonic, and microwave extractions are the generally preferred methods for extraction with ultrasonic- and microwave-assisted extraction discovered to show greater improvements than continuous methods (71). Column chromatography is used for the separation of curcumin from the derivatives of curcuminoids such as demethoxycurcumin and bisdimethoxycurcumin. This is done through the adsorption of the mixture by silica gel (stationary phase) with various solvent mixtures such as methanol/chloroform or chloroform/dichloroform acting as the mobile phase. Methods for detecting curcumin include UV absorption detectors, Liquid Chromatography/Mass Spectrometry, and HPLC. Metabolism, biodistribution, and pharmacokinetics have been assessed by detecting curcumin in biofluids using LC/MS and HPLC.

Ultrasonic extraction can generate a high product yield with a reduction in processing time and less solvent consumption. This increase in the product is due to sound waves being propagated in the solvent and the creation of microcavities, which resulted in the reduction of particle size. Bubbles from the cavities are imploded and this leads to high-velocity liquid circulation, collisions of inter-particle, and micro-turbulence formation that lead to an acceleration in diffusion. Curcumin is easily extracted into the extraction medium by the rupturing of the cell wall due to the penetration of ultrasound (72). The ultrasound extraction of natural products indicated that the increase in the yield through the process could be achieved when ultrasound is used in conjunction with other extraction technologies such as microwave and super-crystal extraction, and solid phase extraction is achieved by combining Molecular Imprinted Polymers (MIP) with ultrasound. Molecular Imprinted Polymers is a synthetic sorbent material that is highly porous and contains cavities that selectively bind these molecules and are used for the identification of a single molecule or a group of related molecules (73).

Garlic contains a large number of bioactive compounds that have been reported for medical benefits. Conventional extraction of bioactive compounds from garlic involved the use of polar or non-polar solvents. Bioactive compounds including allicin and allin are major organosulfur compounds that could be extracted using ethanol or water (74).

Extraction of organosulfur compounds from garlic involved incubation of a powdered mixture containing de-ionized water at 25°C, thereby leading to the formation of organosulfur compounds by enzyme-modulated reactions. Another method of extraction of organosulfur compounds is solid-phase micro-extraction coupled with HPLC or GC which involves the partition of the sample matrix bulk contained in a sealed vial and the stationary phase which is a sorption polymer. The ultrasonic-assisted method for extraction of various organosulfur compounds generated a change in the physical and chemical properties of plant materials through the cavitation effect that lead to plant cell disruption, enhancing the release of extractable compounds and mass transport for easy penetration into the sample matrix, thereby enabling solid phase and liquid phase to maintain a high surface area contact. This method was effective in extracting organosulfur compounds, total phenols, and flavonoids, with all compounds except flavonoids dependent on temperature (75).

Another method for extraction of organosulfur compounds is Dispersive Liquid-Liquid Micro-Extraction (DLLME). This method was applied in response to the trends of using more miniature and greener extraction techniques. Solutes which are hydrophobic in nature are present in large quantities in the extraction solvent and they are dispersed into the bulk aqueous solution. Acetonitrile was used as the dispersive solvent because it is miscible in both aqueous and organic phases while chloroform was used as the extractive solvent due to its higher density than water, and after extraction, the HPLC-UV detector analyzes it. This method reduced analysis time, was more friendly to the environment, and was able to extract higher quality organosulfur compounds from garlic (76). The extraction and analysis of bioactive compounds of garlic using supercritical CO_2_ extraction coupled with supercritical fluid chromatography and mass spectrometry have been carried out (77). This method was generated due to the high lipophilicity of supercritical CO_2_, which inhibited polar compounds' dissolution. The supercritical CO_2_ served as the mobile phase in the chromatographic setup. Methanol was added to the supercritical CO_2_ to enhance the elution capacity of the mobile phase. This method of extraction achieved better separation in less time and bioactive compounds showed acceptable recovery, precision, and sensitivity (78, 79).

## Phytochemicals associated with ginger, turmeric, and garlic

The use of the High-performance liquid chromatographic (HPLC) method has been reported as the usual method of analysis for 6-gingerol, 6-shogaol, 8-gingerol, and 10-gingerol extraction and quantification (80). A recent study shows an alcohol-based deep eutectic solvent of 75% v/v composition, ethanol, and water as a suitable method of extraction with high yields of gingerols in water (81) and indicated that gingerol contents were more from the diluted deep eutectic solvents (DES) than the traditional solvents of ethanol and water.

The different extraction techniques for curcuminoids from turmeric indicated a higher yield from microwave-assisted extraction than the conventional soxhlet extraction (82). Different extraction, isolation, and quantification methods of curcumin and its potential application in respiratory diseases such as COVID-19 were considered. Several methods were found for extraction, isolation, and quantification. But the subcritical water extraction method and HPLC are the most effective method with a higher extraction yield (83).

In the extraction of allicin from garlic (84), studied the three methods of immersion, boiling, and ultrasound using water/ethanol solvents for garlic extraction and compared each other in terms of the extraction speed and time, the antioxidant property of the extract, and the quantity of the heat-sensitive active ingredient. The outcome of the study revealed that ultrasonic extraction is a good alternative to traditional extraction methods. The overview of phytochemicals and their respective bioactive components in ginger, turmeric, and garlic are shown in Table 3.

**Table 3:** The bioactive components in ginger, turmeric, and garlic.

**Plants**	**Phytochemicals**	**Bioactive components**
Ginger	Polyphenols	Gingerols—Shogaols, Zingerone, Paradol (88)
	Phenolics	Quercetin, gingerenone-A, (89)
	Terpenes	β-bisabolene, α-curcumene, zingiberene, α-farnesene, and β-sesquiphellandrene (90)
Tumeric	Antioxidants	Curcumin (70–75%) Bisdemethoxycurcumin (5–10%)
	Polyphenols	Desmethoxycurcumin (10–25%) (29–32)
Garlic	Antioxidants	Allicin, Allin, Ajoene (84)
	Flavonoids	
	Polyphenols	

## Possible modes of action of bioactive compounds in ginger, turmeric, and garlic

Most of the bioactive compounds in the selected plants have antioxidants and anti-inflammatory properties and as such have a good medicinal ability which tends to protect the human body from free radicals by properly neutralizing the radicals (85). They act by protecting against oxidative stress by reducing the reactive oxygen species which are responsible for lipid peroxidation. It is believed that these are the mechanisms of aging because the reactive oxygen species react with organic substances present in the human body (86).

The anti-inflammatory ability of bioactive compounds is *via* suppression of major molecules that are known to play a vital role in cell inflation. Bioactive compounds tend to stop this inflammation, and thereby prevent, inhibit, and downregulate the signal pathway in the cancer model, thereby controlling cancer cells' growth and spread (87).

## Conclusion

The prevalence of existing diseases and the rising cost of treatment/management demands a more sustainable solution with nutraceuticals in view. The bioactive compounds in ginger, garlic, and turmeric such as polyphenolic compounds, organosulfur compounds, vitamins, carotenes, curcumin, and lycopene provided many natural therapeutic benefits. However, the mode of action and effective strategy employed by these compounds are yet to be properly documented. Furthermore, *in-silico* studies and clinical trials involving the use of purified bioactive compounds for therapeutic purposes are important to properly situate their usage as nutraceuticals for biomedical applications.

## Author contributions

CA, OA, PAt, and PAk: manuscript preparation and editing. SA, YO, PAk, and OO: manuscript reviewing. KA: supervision. All authors read and approved the final manuscript.
